# *Borrelia miyamotoi* sensu lato in Père David Deer and *Haemaphysalis longicornis* Ticks

**DOI:** 10.3201/eid2405.171355

**Published:** 2018-05

**Authors:** Yi Yang, Zhangping Yang, Patrick Kelly, Jing Li, Yijun Ren, Chengming Wang

**Affiliations:** Yangzhou University College of Veterinary Medicine, Yangzhou, China (Y. Yang, J. Li, C. Wang);; Jiangsu Co-innovation Center for the Prevention and Control of Important Animal Infectious Diseases and Zoonoses, Yangzhou (Y. Yang, J. Li, C. Wang);; Yangzhou University College of Animal Science and Technology, Yangzhou (Z. Yang);; International Corporation Laboratory of Agriculture and Agricultural Products Safety, Yangzhou University, Yangzhou (Z. Yang);; Ross University School of Veterinary Medicine, Basseterre, St. Kitts and Nevis (P. Kelly);; Dafeng Elk National Natural Reserve, Yancheng, China (Y. Ren);; Auburn University, Auburn, Alabama, USA (C. Wang)

**Keywords:** *Borrelia miyamotoi*, Père David deer, *Haemaphysalis longicornis*, Lyme disease, bacteria, China, extinct in wild, endangered, vector-borne infections, ticks

## Abstract

By sequence analysis of 16S rRNA, *flaB*, *p66*, and *glpQ*, we identified *Borrelia miyamotoi* in 1 of 4 Père David deer (n = 43) seropositive for *Borrelia* spp. and 1.2% (3/244) of *Haemaphysalis longicornis* ticks from Dafeng Elk National Natural Reserve, China. Future studies should assess *Borrelia* pathogenesis in deer.

Père David deer (*Elaphurus davidianus*) are extinct in the wild and found only in captivity, principally in China, England, and the United States. Just 5,000 animals remain, with 40% located in Dafeng Elk National Natural Reserve in China, which attracts >1 million tourists annually. Ticks are common in the Dafeng Elk National Natural Reserve (*1*), so we investigated the tickborne bacterial pathogens in Père David deer at this reserve.

The institutional animal care and use committee of Yangzhou University College of Veterinary Medicine (Yangzhou, China) (YZU-CVM#2015–076) approved this study. We took whole blood samples from 43 apparently healthy Père David deer (20 males, 23 females), separated out the plasma (1,800 × *g* for 10 min), and used the plasma to detect antibodies against bacterial pathogens with the SNAP 4Dx kit (IDEXX, Westbrook, ME, USA) (*2*) according to the manufacturer’s instructions. Further, ELISAs and Western blots using *Borrelia miyamotoi* GlpQ recombinant protein (RayBiotech, Norcross, GA, USA) and peroxidase-labeled rabbit anti-deer IgG (SeraCare, Milford, MA, USA) were performed as described previously (*3*) to detect GlpQ antibodies specific to *B. miyamotoi*.

We collected a convenience sample of *Haemaphysalis longicornis* ticks (n = 244) from elk in the Dafeng Elk National Natural Reserve during the summer of 2016 and stored the collection at −80°C. We used the High Pure PCR Template Preparation Kit (Roche Diagnostics GmbH, Mannheim, Germany) according to the manufacturer’s instructions to extract DNA from Père David deer whole blood samples and entire *H. longicornis* ticks. We used published PCR protocols targeting the 16S rRNA (*4*), *flaB* (*5*), and *glpQ* (*6*) genes and an in-house *p66* PCR (forward primer 5′-CGATTTTTCTATATTTGGACACAT-3′, reverse primer 5′-GATATAGATTCTACAGGTATTGCATAATC-3′) to screen blood samples and ticks for *B. miyamotoi*. We sequenced both strands of PCR products using BGI’s (Shanghai, China) services and aligned them using ClustalW in MEGA 7 (http://www.megasoftware.net/) with the nucleotide sequences of 11 relapsing fever group borreliae and 7 Lyme disease group borreliae found in GenBank.

Four (9.3%; 1 male, 3 females) of the 43 deer were seropositive by SNAP 4Dx, demonstrating an immunodominance of antibodies against synthetic C6 peptide invariable region 6 of the pathogenic *Borrelia* genospecies, *B. burgdorferi* sensu stricto, *B. garinii*, and *B. afzelii* (*7*). Seropositivity was confirmed by GlpQ antibody ELISA and Western blot with GlpQ recombinant protein, indicating exposure to *B. miyamotoi*.

One of the seropositive female deer (2.3% of overall deer population) and 3 (1.2%) of the 244 ticks were positive for the 4 *Borrelia* genes tested (16S rRNA, *flaB*, *glpQ*, *p66*) by PCR. The sequences obtained from the PCR products showed the 4 animals had identical sets of *Borrelia* genes. The 16S rRNA, *flaB*, and *p66* sequences were more similar to those of the relapsing fever group borreliae (16S rRNA 97.9%–99.3%, *flaB* 83.7%–88.9%, *p66* 72.4%–83.3%) than the Lyme disease group borreliae (16S rRNA 96.6%–97.2%, *flaB* 79.2%–80.9%, *p66* 66.1%–68.1%). The *B. miyamotoi* *glpQ* gene sequence obtained from the deer and ticks also clustered with those of the relapsing fever group borreliae (81.1%–88.9%), and all analyzed gene sequences had greatest similarity with *B. miyamotoi* genes (16S rRNA 99.3% [576/580], *flaB* 88.9% [321/361], *p66* 83.3% [423/508], *glpQ* 88.9% [377/424]) (Figure).

**Figure:**
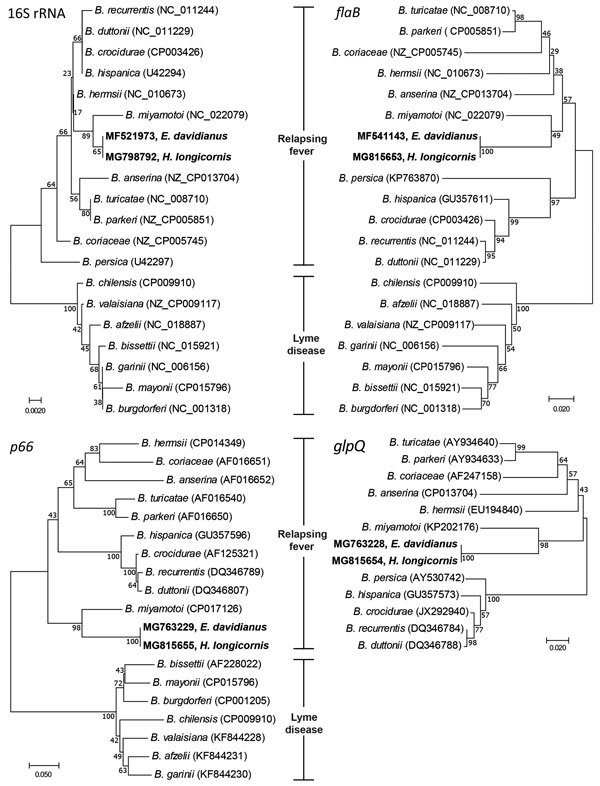
Neighbor-joining phylogenetic trees constructed with 16S rRNA, *flaB*, *p66*, and *glpQ* gene sequences of *Borrelia* spp. isolates collected from Père David deer (*Elaphurus davidianus*) and *Haemaphysalis longicornis* ticks, Dafeng Elk National Natural Reserve, China, and reference isolates. The isolates identified in this study (bold; GenBank accession nos. MF521973, MF541143, MG763228, MG763229) are most similar to *B. miyamotoi* of the relapsing fever group. Numbers at branch nodes show bootstrap support (1,000 replicates). Scale bars indicate nucleotide substitutions per site.

*B. miyamotoi* is a member of the relapsing fever group first isolated in Japan and subsequently found in North America, Europe, and Russia (*8*). *B. miyamotoi* has not been reported in deer but can be pathogenic in humans, usually resulting in an acute febrile influenza-like illness but occasionally causing severe disease, including meningoencephalitis (*9*). Further studies are needed to determine the effects of *B. miyamotoi* infections in deer, especially because studies on *Ixodes scapularis* ticks in the United States have indicated that deer might be a sylvatic reservoir (*10*).

*I. persulcatus* and *I. pavlovskyi* ticks are known to be infected with *B. miyamotoi* in Asia, whereas other *Ixodes* spp. ticks are vectors in the United States and Europe (*9*). Tick control in semi–free-ranging animals is challenging; the Père David deer we studied are commonly infested with ticks. The only tick species identified on Père David deer in Dafeng Elk National Natural Reserve was *H. longicornis* (*1*), which can reach high densities in the environment (summer 89.5 ± 17.1 ticks/10 m^2^, winter 1.47 ± 0.35 ticks/10 m^2^) and cause anemia and even death in heavily infested animals. Our finding of *B. miyamotoi* in *H. longicornis* ticks adds to the list of organisms reported in this tick, primarily *B. burgdorferi* sensu lato and unclassified *Borrelia* spp.

In summary, we have shown that *B. miyamotoi* sensu lato occurs in Père David deer and *H. longicornis* ticks in Dafeng Elk National Natural Reserve. Further studies are needed on the pathogenicity of the organism in deer and the role of *H. longicornis* ticks in the epidemiology of infections in deer and humans.
